# The bii4africa dataset of faunal and floral population intactness estimates across Africa’s major land uses

**DOI:** 10.1038/s41597-023-02832-6

**Published:** 2024-02-12

**Authors:** Hayley S. Clements, Emmanuel Do Linh San, Gareth Hempson, Birthe Linden, Bryan Maritz, Ara Monadjem, Chevonne Reynolds, Frances Siebert, Nicola Stevens, Reinette Biggs, Alta De Vos, Ryan Blanchard, Matthew Child, Karen J. Esler, Maike Hamann, Ty Loft, Belinda Reyers, Odirilwe Selomane, Andrew L. Skowno, Tshegofatso Tshoke, Diarrassouba Abdoulaye, Thierry Aebischer, Jesús Aguirre-Gutiérrez, Graham J. Alexander, Abdullahi H. Ali, David G. Allan, Esther E. Amoako, Samuel Angedakin, Edward Aruna, Nico L. Avenant, Gabriel Badjedjea, Adama Bakayoko, Abraham Bamba-kaya, Michael F. Bates, Paul J. J. Bates, Steven R. Belmain, Emily Bennitt, James Bradley, Chris A. Brewster, Michael B. Brown, Michelle Brown, Josef Bryja, Thomas M. Butynski, Filipe Carvalho, Alan Channing, Colin A. Chapman, Callan Cohen, Marina Cords, Jennifer D. Cramer, Nadine Cronk, Pamela M. K. Cunneyworth, Fredrik Dalerum, Emmanuel Danquah, Harriet T. Davies-Mostert, Andrew D. de Blocq, Yvonne A. De Jong, Terrence C. Demos, Christiane Denys, Chabi A. M. S. Djagoun, Thomas M. Doherty-Bone, Marine Drouilly, Johan T. du Toit, David A. Ehlers Smith, Yvette C. Ehlers Smith, Seth J. Eiseb, Peter J. Fashing, Adam W. Ferguson, José M. Fernández-García, Manfred Finckh, Claude Fischer, Edson Gandiwa, Philippe Gaubert, Jerome Y. Gaugris, Dalton J. Gibbs, Jason S. Gilchrist, Jose M. Gil-Sánchez, Anthony N. Githitho, Peter S. Goodman, Laurent Granjon, J. Paul Grobler, Bonginkosi C. Gumbi, Vaclav Gvozdik, James Harvey, Morgan Hauptfleisch, Firas Hayder, Emmanuel M. Hema, Marna Herbst, Mariano Houngbédji, Brian J. Huntley, Rainer Hutterer, Samuel T. Ivande, Kate Jackson, Gregory F. M. Jongsma, Javier Juste, Blaise Kadjo, Prince K. Kaleme, Edwin Kamugisha, Beth A. Kaplin, Humphrey N. Kato, Christian Kiffner, Duncan M. Kimuyu, Robert M. Kityo, N’goran G. Kouamé, Marcel Kouete T, Aliza le Roux, Alan T. K. Lee, Mervyn C. Lötter, Anne Mette Lykke, Duncan N. MacFadyen, Gacheru P. Macharia, Zimkitha J. K. Madikiza, Themb’alilahlwa A. M. Mahlaba, David Mallon, Mnqobi L. Mamba, Claude Mande, Rob A. Marchant, Robin A. Maritz, Wanda Markotter, Trevor McIntyre, John Measey, Addisu Mekonnen, Paulina Meller, Haemish I. Melville, Kevin Z. Mganga, Michael G. L. Mills, Liaan Minnie, Alain Didier Missoup, Abubakr Mohammad, Nancy N. Moinde, Bakwo Fils E. Moise, Pedro Monterroso, Jennifer F. Moore, Simon Musila, Sedjro Gilles A. Nago, Maganizo W. Namoto, Fatimata Niang, Violaine Nicolas, Jerry B. Nkenku, Evans E. Nkrumah, Gonwouo L. Nono, Mulavwa M. Norbert, Katarzyna Nowak, Benneth C. Obitte, Arnold D. Okoni-Williams, Jonathan Onongo, M. Justin O’Riain, Samuel T. Osinubi, Daniel M. Parker, Francesca Parrini, Mike J. S. Peel, Johannes Penner, Darren W. Pietersen, Andrew J. Plumptre, Damian W. Ponsonby, Stefan Porembski, R. John Power, Frans G. T. Radloff, Ramugondo V. Rambau, Tharmalingam Ramesh, Leigh R. Richards, Mark-Oliver Rödel, Dominic P. Rollinson, Francesco Rovero, Mostafa A. Saleh, Ute Schmiedel, M. Corrie Schoeman, Paul Scholte, Thomas L. Serfass, Julie Teresa Shapiro, Sidney Shema, Stefan J. Siebert, Jasper A. Slingsby, Alexander Sliwa, Hanneline A. Smit-Robinson, Etotepe A. Sogbohossou, Michael J. Somers, Stephen Spawls, Jarryd P. Streicher, Lourens Swanepoel, Iroro Tanshi, Peter J. Taylor, William A. Taylor, Mariska te Beest, Paul T. Telfer, Dave I. Thompson, Elie Tobi, Krystal A. Tolley, Andrew A. Turner, Wayne Twine, Victor Van Cakenberghe, Frederik Van de Perre, Helga van der Merwe, Chris J. G. van Niekerk, Pieter C. V. van Wyk, Jan A. Venter, Luke Verburgt, Geraldine Veron, Susanne Vetter, Maria S. Vorontsova, Thomas C. Wagner, Paul W. Webala, Natalie Weber, Sina M. Weier, Paula A. White, Melissa A. Whitecross, Benjamin J. Wigley, Frank J. Willems, Christiaan W. Winterbach, Galena M. Woodhouse

**Affiliations:** 1 Centre for Sustainability Transitions, Stellenbosch University, Stellenbosch, South Africa; 2 Helsinki Lab of Interdisciplinary Conservation Science, University of Helsinki, Helsinki, Finland; 3 Department of Zoology and Entomology, University of Fort Hare, Alice, South Africa; 4 Centre for African Ecology, School of Animal, Plant and Environmental Sciences, University of the Witwatersrand, Johannesburg, South Africa; 5 Institute of Biodiversity, One Health and Veterinary Medicine, University of Glasgow, Glasgow, Scotland United Kingdom; 6 Chair in Biodiversity Value & Change, Faculty of Science, Engineering & Agriculture, University of Venda, Thohoyandou, South Africa; 7 Department of Biodiversity and Conservation Biology, University of the Western Cape, Bellville, South Africa; 8 Biological Sciences, University of Eswatini, Kwaluseni, Eswatini; 9 Mammal Research Institute, Department of Zoology and Entomology, University of Pretoria, Pretoria, South Africa; 10 School of Animal, Plant and Environmental Sciences, University of the Witwatersrand, Johannesburg, South Africa; 11 Unit for Environmental Sciences and Management, North-West University, Potchefstroom, South Africa; 12 Environmental Change Institute, University of Oxford, Oxford, United Kingdom; 13 Stockholm Resilience Centre, Stockholm University, Stockholm, Sweden; 14 Department of Environmental Sciences, Rhodes University, Makhanda, South Africa; 15 Fynbos Node of the South African Environmental Observation Network, Cape Town, South Africa; 16 South African National Biodiversity Institute, Cape Town, South Africa; 17 Department of Conservation Ecology & Entomology, Stellenbosch University, Stellenbosch, South Africa; 18 Centre for Geography and Environmental Science, University of Exeter, Penryn, Cornwall United Kingdom; 19 School of Geography and the Environment, Environmental Change Institute, University of Oxford, Oxford, United Kingdom; 20 Centre for Environmental Studies, University of Pretoria, Pretoria, South Africa; 21 Department of Agricultural Economics, Extension and Rural Development, University of Pretoria, Pretoria, South Africa; 22 Department of Biological Sciences, University of Cape Town, Cape Town, South Africa; 23 Office Ivoirien des Parcs et Réserves, Soubré, Côte d’Ivoire; 24 African Parks, Bangui, Central African Republic; 25 Environmental Change Institute, School of Geography and the Environment, University of Oxford, Oxford, United Kingdom; 26 Hirola Conservation Programme, Garissa, Kenya; 27 Bird Department, Durban Natural Science Museum, Durban, South Africa; 28 Department of Environment and Sustainability Sciences, University for Development Studies, Tamale, Ghana; 29 Department of Environmental Management, Makerere University, Kampala, Uganda; 30 Biodiversity Conservation, Reptile and Amphibian Program - Sierra Leone, Freetown, Sierra Leone; 31 Department of Mammalogy, National Museum, Bloemfontein, South Africa; 32 Centre for Environmental Management, University of the Free State, Bloemfontein, South Africa; 33 Aquatic Ecology, University of Kisangani/Biodiversity Monitoring Center, Kisangani, Democratic Republic of the Congo; 34 UFR Sciences de la Nature, Universite NanguiI Abrogoua, Abidjan, Côte d’Ivoire; 35 Institut de Recherches Agronomiques et Forestières (IRAF), Centre National de la Recherche Scientifique et Technologique (CENAREST), Libreville, Gabon; 36 Department of Animal and Plant Systematics, National Museum, Bloemfontein, South Africa; 37 Department of Zoology & Entomology, University of the Free State, Bloemfontein, South Africa; 38 Harrison Institute, Sevenoaks, Kent United Kingdom; 39 Agriculture, Health and Environment, Natural Resources Institute, University of Greenwich, Chatham, Maritime United Kingdom; 40 Okavango Research Institute, University of Botswana, Maun, Botswana; 41 Kalahari Research and Conservation, Botswana, Botswana; 42 Independent, Gaborone, Botswana; 43 Giraffe Conservation Foundation, Windhoek, Namibia; 44 Department of Anthropology, University of Minnesota - Twin Cities, Minneapolis, MN USA; 45 Institute of Vertebrate Biology of the Czech Academy of Sciences, Brno, Czech Republic; 46 Eastern Africa Primate Diversity and Conservation Program, Nanyuki, Kenya; 47 BIOPOLIS-CIBIO/InBIO, University of Porto, Porto, Portugal; 48 Biology, Vancouver Island University, Nanaimo, Canada; 49 FitzPatrick Institute of African Ornithology, University of Cape Town, Rondebosch, South Africa; 50 Department of Ecology, Evolution & Environmental Biology, Columbia University, New York, NY USA; 51 ROC USA, Concord, USA; 52 Colobus Conservation, Diani, Kenya; 53 Biodiversity Research Institute (CSIC-UO-PA), Mieres, Spain; 54 Department of Zoology, Stockholm University, Stockholm, Sweden; 55 Department of Wildlife and Range Management, Kwame Nkrumah University of Science and Technology, Kumasi, Ghana; 56 Conserve Global, London, United Kingdom; 57 BirdLife South Africa, Johannesburg, South Africa; 58 Negaunee Integrative Research Center, The Field Museum, Chicago, United States of America; 59 Institut de Systématique, Evolution, Biodiversité (ISYEB), Muséum national d’Histoire naturelle, CNRS, Sorbonne Université, EPHE, Université des Antilles, Paris, France; 60 Faculty of Agronomic Sciences, Laboratory of Applied Ecology, University of Abomey Calavi, Cotonou, Benin; 61 Conservation Programs, Royal Zoological Society of Scotland, Edinburgh, United Kingdom; 62 Institute for Communities and Wildlife in Africa (iCWild), University of Cape Town, Cape Town, South Africa; 63 Centre for Social Science Research (CSSR), University of Cape Town, Cape Town, South Africa; 64 Panthera, New York, USA; 65 Institute of Zoology, Zoological Society of London, London, United Kingdom; 66 Department of Zoology and Entomology, University of Pretoria, Pretoria, South Africa; 67 Centre for Functional Biodiversity, School of Life Sciences, University of KwaZulu-Natal, Pietermaritzburg, South Africa; 68 Ezemvelo KZN Wildlife, Pietermaritzburg, South Africa; 69 Department of Environmental Science, School of Science, University of Namibia, Windhoek, Namibia; 70 Anthropology Department & Environmental Studies Program, California State University Fullerton, Fullerton, United States of America; 71 Gantz Family Collection Center, Field Museum of Natural History, Chicago, USA; 72 Hazi Foundation, Arcaute, Spain; 73 Institute of Plant Science and Microbiology, Universität Hamburg, Hamburg, Germany; 74 Nature Management, University of Applied Sciences of Western Switzerland, Geneva, Jussy Switzerland; 75 Scientific Services, Zimbabwe Parks and Wildlife Management Authority, Harare, Zimbabwe; 76 Laboratoire Evolution et Diversité Biologique, IRD/CNRS/UPS, Université Toulouse III Paul Sabatier, Toulouse, cedex 9 France; 77 Flora Fauna & Man, Ecological Services Limited, Tortola, British Virgin Islands; 78 Independent, Cape Town, South Africa; 79 School of Applied Sciences, Edinburgh Napier University, Edinburgh, Scotland UK; 80 Departamento de Zoología, Universidad de Granada, Granada, Spain; 81 Centre for Biodiversity, National Museums of Kenya, Nairobi, Kenya; 82 Wildlife Conservation Solutions, Mkuze, South Africa; 83 CBGP, IRD, CIRAD, INRAE, Institut Agro, University of Montpellier, Montpellier, France; 84 Genetics, University of the Free State, Bloemfontein, South Africa; 85 Evolution, Ecology, and Organismal Biology, University of California, Riverside, Riverside, CA USA; 86 Department of Zoology, National Museum of the Czech Republic, Prague, Czech Republic; 87 Harvey Ecological, Howick, South Africa; 88 Biodiversity Research Centre, Namibia University of Science and Technology, Windhoek, Namibia; 89 Unité de Formation et de Recherche en Sciences Appliquées et Technologies (UFR-SAT), Université de Dédougou, Dédougou, Burkina Faso; 90 Conservation Services, South African National Parks, Pretoria, South Africa; 91 Organisation pour le Développement Durable et la Biodiversité, Cotonou, Benin; 92 CIBIO-Centro de Investigação em Biodiversidade e Recursos Genéticos, University of Porto, Vairao, Portugal; 93 LIB Museum Koenig, Bonn, Germany; 94 A.P. Leventis Ornithological Research Institute (APLORI), University of Jos, Jos, Nigeria; 95 Biology Department, Whitman College, Walla Walla, WA USA; 96 Department of Natural History, New Brunswick Museum, Saint John, Canada; 97 Evolutionary Biology, Estación Biológica de Doñana (CSIC), Seville, Spain; CIBER, CIBERESP, Madrid, Spain; 98 Natural habitats and biodiversity management, University Félix Houphouet-Boigny, Abidjan, Côte d’Ivoire; 99 Department of Biology, CRSN/ LWIRO, DS Bukavu, DR Congo, Bukavu, Democratic Republic of the Congo; 100 Nature Tanzania, Arusha, Tanzania; 101 Center of Excellence in Biodiversity and Natural Resource Management, University of Rwanda, Huye, Rwanda; 102 Biology, Mbarara University of Science and Technology, Mbarara, Uganda; 103 Department of Human Behavior, Ecology and Culture, Max Planck Institute for Evolutionary Anthropology, Leipzig, Germany; 104 Department of Anthropology, University of California, Los Angeles, USA; 105 Department of Natural Resources, Karatina University, Karatina, Kenya; 106 Zoology, Entomology and Fisheries Sciences, Makerere University, Kampala, Uganda; 107 UFR Environnement, Laboratoire de Biodiversité et Ecologie Tropicale, Université Jean Lorougnon Guédé, Daloa, Côte d’Ivoire; 108 Department of Natural History, Florida Museum of Natural History, University of Florida, Gainesville, USA; 109 Zoology and Entomology, University of the Free State, Qwaqwa campus, Phuthaditjhaba, South Africa; 110 School of Life Sciences, University of KwaZulu-Natal, Scottsville, South Africa; 111 Department of Ecoscience, Aarhus University, Aarhus C, Denmark; 112 Research and Conservation, Oppenheimer Generations, Parktown, Johannesburg, South Africa; 113 Species and Sites Program, Nature Kenya, Nairobi, Kenya; 114 Department of Natural Sciences, Manchester Metropolitan University, Manchester, United Kingdom; 115 Department of Ecology and Wildlife Management, University of Kisangani, Kisangani, Democratic Republic of the Congo; 116 York institute for Tropical Ecosystems, University of York, York, United Kingdom; 117 Conservation Alpha, Cape Town, South Africa; 118 Department of Microbiology and Plant Pathology, University of Pretoria, Pretoria, South Africa; 119 Department of Life and Consumer Sciences, University of South Africa, Roodepoort, South Africa; 120 Centre for Invasion Biology, Department of Botany and Zoology, Stellenbosch University, Stellenbosch, South Africa; 121 Centre for Invasion Biology, Institute of Biodiversity, Yunnan University, Kunming, UMR7179 China; 122 MECADEV CNRS/MNHN, Département Adaptations du Vivant, Muséum National d’Histoire Naturelle, Bâtiment d’Anatomie Comparée, Paris, France; 123 Department of Anthropology and Archaeology, University of Calgary, Calgary, Canada; 124 Department of Environmental Sciences, University of South Africa, Florida, South Africa; 125 Copernicus Institute of Sustainable Development, Utrecht University, Utrecht, The Netherlands; 126 School of Biology and Environmental Science, University of Mpumalanga, Mbombela, South Africa; 127 Centre for African Conservation Ecology, Nelson Mandela University, Gqeberha, South Africa; 128 Faculty of Science, Laboratory of Biology and Physiology of Animal Organisms, Zoology Unit, University of Douala, Douala, Cameroon; 129 Researcher, Conflict and Environmental Observatory, Manchester, United Kingdom; 130 Conservation Biology, Institute of Primate Research-National Museums of Kenya, Nairobi, Kenya; 131 Environmental Sciences, University of Ebolowa, Ebolowa, Cameroon; 132 Wildlife Conservation Ecology Research Group, CIBIO/InBIO, Centro de Investigação em Biodiversidade e Recursos Genéticos, Universidade do Porto, Vairã, Portugal; 133 BIOPOLIS Program in Genomics, Biodiversity and Land Planning, CIBIO, Campus de Vairão, Vairão, Portugal; 134 African Parks, Johannesburg, South Africa; 135 Moore Ecological Analysis and Management, LLC, Gainesville, USA; 136 Mammalogy Section-Department of Zoology, National Museums of Kenya, Nairobi, Kenya; 137 Laboratoire d’Ecologie, de Botanique et de Biologie végétale, University of Parakou, Parakou, Benin; 138 Indigenous Woodland Strategy Area, Forestry Research Institute of Malawi, Zomba, Malawi; 139 Institute of Environmental Sciences, Faculty of Technology and Sciences, University Cheikh Anta Diop de Dakar, Dakar, Sénégal; 140 Institut de Systématique, Evolution, Biodiversité (ISYEB), Muséum national d’Histoire naturelle, CNRS, Sorbonne Université, EPHE, Université des Antilles, Paris, France; 141 Departement of Biology, Faculty of Science, University of Kinshasa, Kinshasa, Democratic Republic of the Congo; 142 Department of Animal Biologie and Physiologie, University of Yaounde I, Yaounde, Cameroon; 143 Primatology, Center for Research in Ecology and Forestry (CREF), Bikoro, Democratic Republic of the Congo; 144 Białowieża Geobotanical Station, Faculty of Biology, University of Warsaw, Białowieża, Poland; 145 Small Mammal Conservation Organization, Benin City, Nigeria; 146 Biological Sciences, Texas Tech University, Lubbock, United States of America; 147 Department of Biological Sciences, University of Sierra Leone, Freetown, Sierra Leone; 148 Nature Uganda, Kampala, Uganda; 149 Institute for Communities and Wildlife in Africa, University of Cape Town, Cape Town, South Africa; 150 Animal Production Institute, Rangeland Ecology, Agricultural Research Council, Pretoria, South Africa; 151 College of Agriculture and Environmental Sciences: Department of Environmental Sciences (ABEERU), University of South Africa, Pretoria, South Africa; 152 Frogs & Friends, Berlin, Germany; 153 Chair of Wildlife Ecology & Management, University of Freiburg, Freiburg, Germany; 154 KBA Secretariat, c/o BirdLife International, Cambridge, United Kingdom; 155 Institute of Biosciences, Department of Botany, University of Rostock, Rostock, Germany; 156 Department of Economic Development, Environment, Conservation & Tourism, North West Provincial Government, Mahikeng, South Africa; 157 Department of Conservation and Marine Sciences, Cape Peninsula University of Technology, Cape Town, South Africa; 158 Department of Botany and Zoology, Stellenbosch University, Stellenbosch, South Africa; 159 Division of Conservation Ecology, Sálim Ali Centre for Ornithology and Natural History, Coimbatore, India; 160 Mammalogy Department, Durban Natural Science Museum, Durban, South Africa; 161 Herpetology, Museum für Naturkunde - Leibniz Institute for Evolution and Biodiversity Science, Berlin, Germany; 162 Department of Biology, University of Florence, Sesto Fiorentino, Italy; 163 Department of Zoology, Al-Azhar University, Cairo, Egypt; 164 School of Life Sciences, University of KwaZulu Natal, Durban, South Africa; 165 Gesellschaft fuer Internationale Zusammenarbeit (GIZ), Addis Ababa, Ethiopia; 166 Department of Biology and Natural Resources, Frostburg State University, Frostburg, USA; 167 CIRI, Centre International de Recherche en Infectiologie, Université de Lyon, Lyon, France; 168 Ornithology Section, Zoology Department, National Museums of Kenya, Nairobi, Kenya; 169 Biological Sciences and Centre for Statistics in Ecology, Environment and Conservation, University of Cape Town, Cape Town, South Africa; 170 Cologne Zoo, Cologne, Germany; 171 Conservation Division, BirdLife South Africa, Johannesburg, South Africa; 172 Applied Behavioural Ecological & Ecosystem Research Unit (ABEERU), University of South Africa, Florida, South Africa; 173 Laboratory of Applied Ecology, University of Abomey-Calavi, Abomey-Calavi, Benin; 174 Mammal Research Institute, Centre for Invasion Biology, Department of Zoology and Entomology, University of Pretoria, Pretoria, South Africa; 175 Independent researcher, London, United Kingdom; 176 Department of Biology, University of Venda, Thohoyandou, South Africa; 177 Biology, University of Washington, Seattle, USA; 178 Endangered Wildlife Trust, Midrand, South Africa; 179 Grasslands-Forests-Wetlands Node of the South African Environmental Observation Network, Pietermaritzburg, South Africa; 180 Wildlife Conservation Society, Bronx, USA; 181 Ndlovu Node of the South African Environmental Observation Network, Phalaborwa, South Africa; 182 Gabon Biodiversity Program, Smithsonian National Zoo and Conservation Biology Institute, Center for Conservation and Sustainability, Gamba, Gabon; 183 Biodiversity Capabilities Directorate, CapeNature, Cape Town, South Africa; 184 Department of Biodiversity and Conservation Biology, University of the Western Cape, Cape Town, South Africa; 185 FunMorph Lab, Department of Biology, University of Antwerp, Antwerp, Belgium; 186 AfricanBats NPC, Centurion, South Africa; 187 Evolutionary Ecology Group, University of Antwerp, Wilrijk, Belgium; 188 Arid Lands Node of the South African Environmental Observation Network, Kimberley, South Africa; 189 NWU Botanical Garden, School of Biological Sciences, North-West University, Potchefstroom, South Africa; 190 Richtersveld Desert Botanical Gardens, Richtersveld National Park, SANParks, Sendelingsdrift, South Africa; 191 Department of Conservation Management, Nelson Mandela University, George, South Africa; 192 Institut de Systématique, Evolution, Biodiversité, Muséum National d’Histoire Naturelle, Paris, France; 193 Department of Botany, Rhodes University, Makhanda, South Africa; 194 Accelerated Taxonomy, Royal Botanic Gardens, Kew, Richmond, United Kingdom; 195 Restoration Ecology, Technische Universität München, Freising, Germany; 196 Department of Forestry and Wildlife Management, Maasai Mara University, Narok, Kenya; 197 Department of Migration, Max Planck Institute of Animal Behavior, Radolfzell, Germany; 198 Ecological Consultant, Fürth, Germany; 199 SARChI (NRF-DST) Research Chair on Biodiversity Value and Change, University of Venda, Thohoyandou, South Africa; 200 Center for Tropical Research, Institute of the Environment and Sustainability, University of California Los Angeles, Los Angeles, USA; 201 Landscape Conservation Programme, BirdLife South Africa, Johannesburg, South Africa; 202 Plant Ecology, University of Bayreuth, Bayreuth, Germany; 203 School of Natural Resource Management, Nelson Mandela University, George, South Africa; 204 Scientific Services, South African National Parks, Skukuza, South Africa; 205 Kigelia Solutions, Lusaka, Zambia; 206 Tau Consultants (Pty) Ltd, Maun, Botswana; 207 University of Edinburgh, Edinburgh, Scotland UK

**Keywords:** Biodiversity, Conservation biology, Restoration ecology, Plant ecology, Agroecology

## Abstract

Sub-Saharan Africa is under-represented in global biodiversity datasets, particularly regarding the impact of land use on species’ population abundances. Drawing on recent advances in expert elicitation to ensure data consistency, 200 experts were convened using a modified-Delphi process to estimate ‘intactness scores’: the remaining proportion of an ‘intact’ reference population of a species group in a particular land use, on a scale from 0 (no remaining individuals) to 1 (same abundance as the reference) and, in rare cases, to 2 (populations that thrive in human-modified landscapes). The resulting bii4africa dataset contains intactness scores representing terrestrial vertebrates (tetrapods: ±5,400 amphibians, reptiles, birds, mammals) and vascular plants (±45,000 forbs, graminoids, trees, shrubs) in sub-Saharan Africa across the region’s major land uses (urban, cropland, rangeland, plantation, protected, etc.) and intensities (e.g., large-scale vs smallholder cropland). This dataset was co-produced as part of the Biodiversity Intactness Index for Africa Project. Additional uses include assessing ecosystem condition; rectifying geographic/taxonomic biases in global biodiversity indicators and maps; and informing the Red List of Ecosystems.

## Background & Summary

Accelerating socio-economic development over the past century has caused a dramatic transformation of ecosystems, through human activities such as cultivation, urbanisation, resource extraction and infrastructure development^1,2^. Human land use activities are major drivers of biodiversity loss^3,4^. As awareness grows about the scale and pace of biodiversity loss, so does our understanding of the importance of biodiversity to human well-being^5^. Despite this increased awareness, development agendas and policy interventions persistently overlook the critical support-system role of biodiversity in sustainable development and as a source of resilience in times of change^6–10^. This under-representation is perpetuated by the scarcity of suitable biodiversity data and the difficulty of consistently quantifying biodiversity at the scales relevant for policy, in metrics that indicate its support-system role and the impacts of human activities on that support system^10–12^.

Existing biodiversity datasets that could be used to assess human impacts on biodiversity and the support system it provides have significant limitations that hamper the mainstreaming of biodiversity into policy and planning. Firstly, these datasets are biased across taxa (towards larger, more conspicuous species, especially large mammals and birds^13–15^), regions (towards North America and Europe, with Africa being particularly under-represented^13–15^), and land uses (towards more intact land uses, notably protected areas^14,16^). Secondly, to consistently assess and compare anthropogenic impacts on biodiversity, the current state of biodiversity ideally needs to be compared to a reference state^17^. This comparison requires biodiversity data to be collected using comparable methods either over time (e.g., McRae *et al*.^16^) or across contemporary human-modified and unmodified ‘intact’ landscapes (e.g., Schipper *et al*.^18^), further curtailing data availability. Finally, the connection between common biodiversity metrics (e.g., global or regional threat status of species; representation of species distributions within protected areas^14,19^) and the functional dimensions of biodiversity relevant to its support-system roles is often unclear^6,10^. Such roles typically manifest at local scales and depend on the population abundance of species within different functional groups, and how they are impacted by human land uses in these areas^11,20,21^. Exemplifying these limitations, the largest global dataset of species population abundances across different land uses (the ‘PREDICTS’ database^22^) includes just 35 Afrotropical studies with population counts in an anthropogenically modified site compared with an ‘intact’ reference site. African decision-makers have noted data limitations as a major constraint to mainstreaming biodiversity into national sustainable development efforts^23^, and such limitations can bias international decision-making towards Global North solutions^24^.

**Fig. 1:**
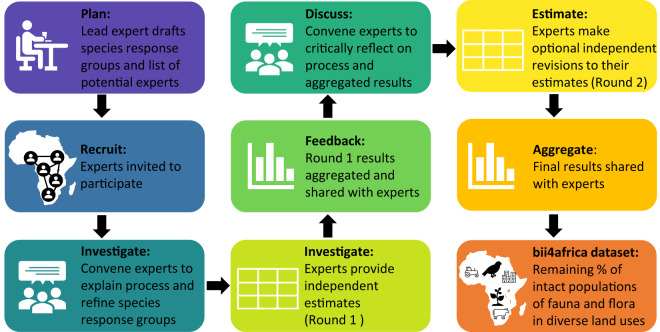
Overview of the structured expert elicitation process, based on the IDEA protocol (Investigate, Discuss, Estimate, Aggregate). This process was run for each broad taxonomic group (Table 1), between November 2020 and January 2022, to elicit from 200 experts the estimated impact of nine major African land uses (Table 2) on the relative population abundances of terrestrial vertebrates and vascular plants.

We address this data gap through a structured expert elicitation process (Fig. 1) involving 200 experts in Afrotropical biodiversity on mainland sub-Saharan Africa. Expert elicitation is used widely in conservation and natural resource management when data are insufficient or absent^25^. Notable examples of where expert elicitation has been used include the International Union for Conservation of Nature (IUCN) Red List of Threatened Species^26^, and assessments of the Intergovernmental Panels on Climate Change^27^ and on Biodiversity and Ecosystem Services^5^. We employed the latest advances in expert elicitation to ensure data rigour and consistency^25^. Expanding substantially on an earlier approach that involved 16 experts in Southern Africa^28^, experts estimated ‘intactness scores’: the remaining proportion of an ‘intact’ reference population of a given species group (Table 1) in a particular land use (Table 2), on a scale from 0 (no remaining individuals) to 1 (same abundance as a reference population) and, in rare cases, 2 (populations that thrive in human-modified landscapes). The ‘intact’ reference is the population abundance that would likely have occurred in the area before alteration by modern industrial society. Because information on species populations from this era is virtually non-existent, standard protocol is to consider a remote wilderness area with a natural disturbance regime^28^ (a Hybrid-Historical approach^17^) where necessary. The resulting bii4africa dataset contains standardised intactness scores representing terrestrial vertebrates (tetrapods: ±5,400 amphibians, reptiles, birds and mammals) and vascular plants (±45,000 forbs, graminoids, trees and shrubs) in sub-Saharan Africa across nine major land uses of varying intensity.

**Table 1:** Broad taxonomic groups encompassing all sub-Saharan African terrestrial vertebrates and vascular plants, with rationale for how they were allocated into species response groups.

Taxonomic group	Response group allocation
Birds (Aves) - *1970 species* *- 17 response groups*	Large, taxonomically unique species were allocated into response groups first: vultures and raptors (large and small), large terrestrial species (e.g., cranes) and waterbirds. Two additional wide-ranging groups were added: aerial feeders (swifts and swallows) and opportunistic species that thrive in human-dominated landscapes. The remaining groups were classified based on their habitat nesting location (cavity, ground, other) and body size.
Amphibians (Amphibia) - *799 species* *- 7 response groups*	Experts emphasised the influence of breeding habitat in how amphibians respond to human-modified landscapes. Six groups were thus defined by the predominant habitat in which each species breeds (direct developers, water: permanent or ephemeral and flowing or still, seep, tree hollow).
Reptiles (Reptilia) - *1481 species* *- 13 response groups*	Habitat utilisation, taxonomy, and body size (in a hierarchical manner) influence likely responses of reptiles. First, species occupying strictly aquatic, fossorial, rupicolous, or arboreal habitats were grouped. Because of their derived life history traits, the remaining chelonians were allocated to their own group. Remaining species were grouped on the basis of taxonomy (snake vs lizard), body size, and degree of habitat specialisation.
Mammals (Mammalia)	
Bats - *214 species* *- 10 response groups*	Bats were grouped by their foraging strategy (clutter, edge or open environment insect foragers; fruit-eaters), their roosting location (cave, crevice, foliage) and degree of roost flexibility.
Insectivores - *205 species* *- 9 response groups*	Insectivores were grouped by a combination of taxonomy, body size, habitat (forest, montane, savanna) and stratum (aquatic, arboreal, fossorial, terrestrial).
Rodents, rabbits, hyraxes - *426 species* *- 16 response groups*	Rodents were grouped based on a combination of their taxonomy, habitat preference (arid, forest, montane, savanna/grassland, wetland), body size, diet (herbivorous, granivorous, generalist), stratum (aquatic, arboreal, fossorial, rupicolous, terrestrial) and habits (diurnal, nocturnal).
Small carnivores, aardvark, pangolins - *70 species* *- 16 response groups*	Small carnivores were grouped based on range size (esp. when restricted vs large); stratum (arboreal, terrestrial, aquatic); habitat(s) (notably to differentiate forest, savanna, rocky); diet (esp. for specialised vs omnivorous species); foraging socio-ecology (solitary vs social); body size; and taxonomic affiliation. Five non-carnivoran mammalian species were also included because of similar ecological characteristics, relatively small body size, and similar phylogenetic relationships.
Large carnivores - *7 species*	The elicitation was done at species level; no response groups were provided.
Large herbivores - *95 species*	The elicitation was done at species level; with each large mammal herbivore species also allocated into one of 12 response groups based on diet (grazer, browser, mixed, frugivore) and body size, with sociality, habitat (e.g., arid, montane, water-dependent), movement (e.g., migratory) and taxonomic groupings also considered.
Primates - *106 species* *- 6 response groups*	Primates were grouped using a combination of habitat (primary/secondary rainforest, woodland/savanna, grassland, generalist found in moist and more arid forests/woodlands); stratum (strictly arboreal, semi-terrestrial, terrestrial); and diet (omnivorous, specialist).
Vascular plants: graminoids, forbs, trees and shrubs *~45,000 species* *- 33 response groups*	Plants were first differentiated by growth form: graminoids (grasses, sedges, rushes); forbs (herbaceous annuals and perennials, geophytes, geoxyles, tubers, herbaceous climbers, dwarf shrubs, succulents, suffrutices); and trees and shrubs (including woody lianas and epiphytes). Within each of these broad groups, plants were grouped based on an assembly of traits that facilitate survival by avoiding, promoting, resisting or tolerating dominant abiotic or biotic limitations to growth (e.g., water, light, herbivory, fire). The potential for humans to modify these abiotic and biotic drivers contributed to the decision to assess plant groups within biomes. E.g., deforestation for agriculture removes light competition constraints, potentially allowing an influx of savanna grasses. By contrast, clearing a savanna environment for agriculture would have much less effect on light availability, and different shifts in plant groups would be anticipated.

An expert elicitation process was run for each broad taxonomic group, with participants estimating intactness scores for each response group. Supplementary Table 1 includes details of each response group.

**Table 2:** Descriptions of the nine sub-Saharan African land uses presented to experts to estimate the remaining proportion of an ‘intact’ reference population, for diverse groups of species.

Land use	Description
Dense urban	Densely built-up environments with high human population densities and limited green space – city centres, dense townships, industrial areas, transformed mining areas (e.g., open cast mines, quarries, dumps). Most ecological processes are highly modified. There are few remaining near-natural patches in the landscape, except for e.g., road-side trees, small parks.
Mixed settlements	Suburban areas, smaller towns and rural settlements with large but fragmented human populations interspersed with gardens, parks and near-natural patches of open space, potentially with low densities of cattle, goats, sheep or chickens, or small-scale croplands.
Non-intensive smallholder croplands	Lands used mainly for smallholder agriculture in small fields (<2 ha), consisting of a diversity of short-duration and long-duration crops (e.g., maize, millet, cassava, beans, squashes, as well as scattered fruit, shade or timber trees). Agricultural inputs of fertilisers and pesticides are very low if any, cultivation is usually manual, there is little or no ploughing or irrigation, and harvest is staggered in time. Fields and homesteads are interspersed with patches of near-natural vegetation. These lands often also support low densities of livestock or smallstock, which are partly free-roaming, and may have semi-natural grazing areas in addition to eating crop residues and cut forage.
Intensive large-scale croplands	Lands used mainly for short duration, monocultural crops in large fields (e.g., staple cereal crops, soybeans, sugar cane). Land use activities usually include several of the following: annual ploughing, inorganic fertiliser application, pesticide application, irrigation, mechanisation. When the crop is harvested, the entire biomass is removed and the next crop is planted, perhaps after a fallow period. There are few remaining near-natural patches in the landscape, except for instance on drainage lines, field boundaries and contour strips, or some woodlots or windbreaks of trees.
Tree crop (fruit) plantations	Lands used mainly for tree crops including fruit-bearing tree or shrub plantations (e.g., bananas, coffee, oil palm, cacao, oranges, vineyards, nuts). Non-transformational harvest, usually only the fruit is taken, and trees may be replaced at some stage. Includes limited remnant forest, riparian or grassland patches between plantation compartments.
Timber plantations	Lands used for growing trees, typically exotic species, for saw timber, poles or pulp. Harvested by clear-cut every 10 to 30 years, and replanted or regrown from coppice. Includes limited remnant forest, riparian or grassland patches between plantation compartments.
Intensive rangelands	Lands used mainly for livestock grazing either with input of fertiliser or pesticide, or with high stock density relative to what the land can sustain (high enough to cause some disturbance or to stop regeneration of vegetation, or to have done so in the recent past). Domesticated stock such as cattle, sheep, goats are typical, but could also include intensive use of indigenous species such as ostrich.
Near-natural lands	Lands (which could be forests, savannas, arid lands, mountainous lands, grasslands) remote from infrastructure, having only minor transformational land use such as crops, planted trees, livestock and human settlements. The human population is relatively low, and livestock or crop-based agriculture or harvest of resources is not at levels that substantially alter natural ecological processes or habitats.
Strictly protected areas	Strictly protected areas that generally do not allow for permanent settlements or resource use, though sometimes allow tourism including limited accommodation and road infrastructure (World Database on Protected Area categories I-III or equivalent). Minimal recent human impact on structure, composition and function of the ecosystem.

Supplementary Table 2 includes representative images of each land use.

The dataset was developed to enable the quantification of the Biodiversity Intactness Index^28^ for sub-Saharan Africa (https://bii4africa.org/). It provides an African-led alternative to a previous attempt to map the Biodiversity Intactness Index globally based on a model^29^, which produced inaccurate results for Africa^30^ (likely in part because of the under-representation of African data in the model). The bii4africa dataset that we present here has a broad range of additional uses, including assessing and mapping ecosystem condition to inform national planning and reporting on Goal A in the post-2020 Global Biodiversity Framework; alleviating geographic and taxonomic biases in global biodiversity indicators and maps; parameterising models of biodiversity in a changing world; informing the IUCN Red List of Ecosystems; supporting the United Nations Decade on Ecosystem Restoration in identifying priority ecosystems and monitoring the impact of investments into restoration; identifying the properties of novel ecosystems; and informing future research and training in African biodiversity (Table 3). Importantly, the dataset—co-produced by 200 experts—embodies context-specific knowledge on African biodiversity that contributes to inclusivity in ecology^31^. It is also a positive response to the recent call by the Intergovernmental Science-Policy Platform on Biodiversity and Ecosystem Services (IPBES) for African-led research that closes knowledge gaps by mobilising local data^24^.

**Table 3:** A non-exhaustive list of potential uses of the bii4africa dataset.

Use	Details
Quantifying ecosystem integrity/condition across space and through time	The first goal of the post-2020 Global Biodiversity Framework is to increase the ‘area, connectivity and integrity of natural ecosystems’. The dataset can be used towards assessing ecosystem condition, e.g., mapping the Biodiversity Intactness Index^28^.
Assessing the severity of functional decline for the IUCN Red List of Ecosystems	Aggregated indices of ecosystem health or condition are proposed as one option for quantifying functional decline for the IUCN Red List of Ecosystems^54^. The dataset could enable such a quantification.
Quantifying relative population abundance and biodiversity composition indicators	This dataset could be used towards quantifying several composite biodiversity indicators, e.g., Essential Biodiversity Variables^55^; Multidimensional Biodiversity Index^12^; Ecosystem Integrity Index^56,57^; Biodiversity Intactness Index^28,29^; and Mean Species Abundance metric (GLOBIO)^18,43^. Several of these are also proposed indicators in the Global Biodiversity Framework.
Setting conservation and restoration goals and/or monitoring progress towards these goals	The dataset could be used to assess progress towards restoring ‘intactness’ in a region. The data could also be used in prioritisation exercises to identify ecosystems for restoration action to maximise improvements in biodiversity intactness.
Assessing the impact of regional development plans	Large-scale infrastructure and agriculture projects are planned across sub-Saharan Africa (e.g., Laurance *et al*.^58^). This dataset could be used to predict the impacts of such development plans on biodiversity intactness.
Considering biodiversity sensitivity to development	The data could identify the types of taxa that are particularly sensitive to development, to inform Environmental Impact Assessments and other development plans.
Identifying indicator species groups	Species groups with lower intactness scores are more vulnerable to environmental or developmental change, and monitoring their populations could give early warnings of system degradation.
Assessing trends in how diverse species respond to land use activities	The data could be analysed to test hypotheses and explore trends across species groups and/or land uses.
Species ecological (as opposed to taxonomic) classifications	The species response groups presented in this dataset (Supplementary Table 1) may be useful for a range of applications that require species to be organised into ‘functional’ (as opposed to purely taxonomic) categories.
Zoonotic disease risk and mitigation assessments	The dataset could be used in identifying and monitoring species groups (and areas, if spatialised) to prevent zoonotic and epizootic disease outbreaks.
Characterising novel ecosystems	Intactness scores >1 depict species groups that respond positively to human land use activities, thus contributing to understanding novel ecosystems^59^.
Parameterising, calibrating and validating models of biodiversity in a changing world	Biodiversity models are used to predict biodiversity patterns across space and through time (e.g., Di Marco *et al*.^60^; Harfoot *et al*.^61^; Schipper *et al*.^18^) under changing land use conditions. This dataset could be used to parameterise, calibrate, or validate such models.
Climate change research	The approach taken in this paper offers opportunities for natural and/or experimental designs to test interactions of biodiversity, land use and climate change across variable spatial and temporal scales.
Informing future research and training in biodiversity	The species groups and land uses for which there were either few scores or large expert score variability highlight knowledge gaps that require further study. These knowledge gaps could also be used to guide future scientist training efforts.
Comparison with other regions, taxonomic groups or time periods	A similar expert-elicited approach could be used to estimate intactness scores for other regions/taxonomic groups (e.g., invertebrates), allowing for comparison with this dataset. The approach could be repeated in the future to assess how knowledge on land use impacts on biodiversity abundance has changed.
List of biodiversity experts to contact for data, collaboration, etc.	The 200 participating experts (see author list and contributions, and https://bii4africa.org/category/experts/) can serve as points of contact for global initiatives looking to aggregate data or build collaborations.

Supplementary Table 3 includes further details on these uses.

## Methods

### Elicitation planning and expert recruitment

We implemented a published, modified-Delphi protocol (multiple rounds of individual, independent expert estimation interspersed with group discussion and review; Fig. 1), which has been shown to improve the rigour of elicitation outcomes^25^. The elicitation was limited to terrestrial vertebrates (tetrapods) and vascular plants – groups that comprise species with a high diversity of attributes and functions. Experts were sought with knowledge of the degree to which human land uses impact populations of these species groups in sub-Saharan Africa (or a region therein). Such expert knowledge is typically limited to one taxonomic class (e.g., birds or reptiles), or in the case of mammals, often just one or several orders (e.g., primates or bats). As such, multiple expert elicitation processes were run between November 2020 and January 2022, to cover the various broad taxonomic groups of species (see Table 1). An expert in each broad taxonomic group was invited to lead the elicitation for that taxonomic group. Each ‘lead expert’ was identified based on their relevant expertise and existing network across the continent or willingness to develop such a network. For example, several lead experts serve in the IUCN Species Survival Commission working groups and other regional networks (e.g., https://ascaris.org/; https://www.birdlife.org/our-partners-africa/). The lead expert was responsible for identifying experts and inviting them to participate, as well as proposing a draft list of species response groups and providing input into the development of the land use categories. These tasks are detailed in the subsequent sections.

#### Identifying and recruiting experts

A broad definition of expertise was used to identify experts, centred on experience of how sub-Saharan species are impacted by human land uses^25,32^. Diverse types of people can have such experience (e.g., researchers, field or tour guides, park rangers, conservation practitioners, museum curators, and consultants), and inclusion was thus not limited to specific qualifications or institutional affiliations. The aim was to include about 20 experts for each broad taxonomic group, according to guidelines based on practicality and evidence of limited improvements in group performance above 6 to 12 experts^25^.

The lead expert identified individuals known to have relevant expertise. If this activity did not achieve the target of 20 individuals, additional experts were identified through relevant publications (using appropriate search terms on Google Scholar) and websites (e.g., specialist nature guides or tours, conservation organisations). In some cases, participating experts were asked to recommend other experts (snowball sampling). An invitation was emailed by the lead expert to each identified expert, explaining the project and what would be required of them. Experts confirmed that they had the relevant experience and were willing to participate by returning a signed consent form, including demographic details (notably taxa, years and region of experience). Ethical clearance for the project was provided by Stellenbosch University (project number 15182).

**Fig. 2:**
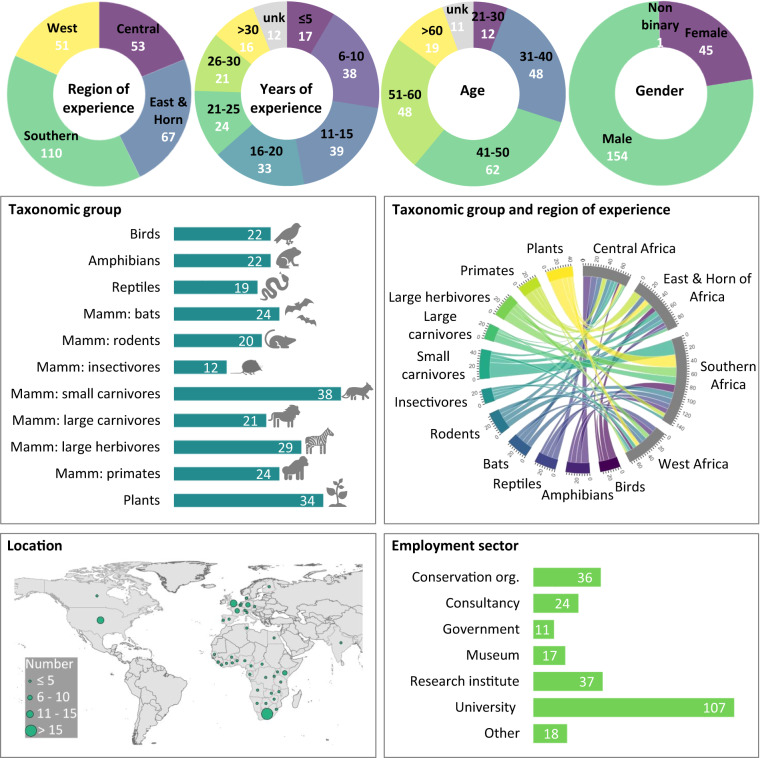
Attributes of the 200 participating experts. All values in white font (and black font on the cord plot) represent the number of experts. Numbers do not add up to 200 when categories are not mutually exclusive (region, taxonomic group, employment sector) or when experts did not report a certain attribute (unk = unknown; Mamm = Mammals; org. = organisation).

Of the 248 experts who agreed to participate, a total of 200 (81%) participated in the full elicitation process (https://bii4africa.org/category/experts/), with an average of 24 experts per broad taxonomic group (Fig. 2). These experts were from 39 countries, including 23 African countries (59% of experts were African nationals). Most experts (72%) were resident in Africa at the time of elicitation, across 26 countries. Their experience spanned sub-Saharan Africa, with Southern Africa being best represented, followed by East Africa, and West Africa having the lowest representation. Experts worked in a range of sectors, most commonly at universities, followed by research institutes and conservation organisations. Collectively, experts had over 3,300 years of relevant accumulated experience, with a mean (±SD) of 18 ± 10 years per expert.

#### Identifying and describing species response groups

It was not possible for experts to estimate the impact of different human land uses on the abundance of every terrestrial vertebrate and vascular plant species in sub-Saharan Africa (±50,000 species), both because of the large number of estimates that would be required, and because there are many species for which there is limited ecological knowledge. Therefore, species that were expected to respond in a similar way to human land uses were assigned to a ‘species response group’^28^. These groups can be considered broadly synonymous to functional groups^33^, though our focus is specifically on common responses of population abundance to human land use.

Within each broad taxonomic group (Table 1), the lead expert proposed a draft set of species response groups, based on their knowledge of the key organismal attributes likely to determine the impact of different land uses on populations. These draft species response groups were presented to the participating experts for that taxonomic group during an introductory planning meeting (see *Structured expert elicitation* section below) and revised based on experts’ feedback. Key organismal attributes that informed species response group categorisation varied between the taxonomic groups (Table 1; Supplementary Table 1). For terrestrial vertebrates, attributes commonly included habitat requirements (e.g., forest, grassland, generalist), body size, diet and stratum (e.g., arboreal, fossorial, rupicolous). Terrestrial vascular plants were divided into three broad groups: trees and shrubs, graminoids, and forbs, and species response groups were described within each broad group based on a framework of life-history strategies shaped by disturbance^34^. Species response groups were thus ecologically defined, not necessarily taxonomically defined. Large mammals were an exception to the response-group approach. The elicitation was done at species level for the seven large carnivores and 95 large herbivores. While the large herbivores were initially categorised into response groups, experts decided during the introductory meeting to estimate per species rather than per species response group. Thus, for large herbivores, we present species response groups, as well as species-level estimates. Details of each response group are included in the ‘Sp_Groups’ spreadsheet in the bii4africa dataset^35^.

For each broad taxonomic group of terrestrial vertebrates, the lead expert allocated each IUCN-listed species (up to date at the time of the elicitation) in sub-Saharan Africa into the appropriate species response group, with input from participating experts where necessary (‘Sp_Vert’ spreadsheet in the bii4africa dataset^35^). Determining an average intactness score across species for a given region (country, ecoregion, biome, etc.) can thus be done including only the species that occur in that region, based on species range maps available through the IUCN Red List^26^. By contrast, allocating each plant species into its respective response group was not possible given the large number of species (~45,000). Because of this limitation, a biogeographical delineation was included in the elicitation process. Experts were asked to provide estimates for the plant species response groups in eight biogeographical units (forest, Caesalpinioid-miombo humid savanna, mixed-acacia savanna, grassland, shrubland, thicket, desert and fynbos). These represent the major sub-Saharan African biomes, with savanna—the most extensive biome—treated as two biomes differentiated by distinct vegetation types. This delineation ensured that only plants present in each biome were considered in the estimation process and in any subsequent data aggregations. Because biome-specific environmental and evolutionary assembly processes act as a strong filter to the set of ‘available traits’, not all plant response groups occur in all biomes (e.g., there are no fire-tolerant forbs in the forest biome). Plant experts were also asked to estimate, within the broad groups of trees and shrubs, forbs and graminoids, the proportion of species within each species response group in each biome (‘Sp_Plant’ spreadsheet in the bii4africa dataset^35^). These estimates provide a proxy for the distribution of species richness across the plant groups in each biome (e.g., the proportional richness of the graminoid response groups in the desert biome sums to 1). To enable data users to consistently spatialise these biomes, each ecoregion (based on Ecoregions2017© Resolve map^36^) was allocated to one (or two, if considered a mosaic) of these eight biomes informed by the literature^37–39^ and expert opinion (‘Biome’ spreadsheet in the bii4africa dataset^35^).

#### Identifying and describing land use categories

Intactness scores were estimated for nine distinct land uses (Table 2). These land uses with varying intensities of human modification were selected to capture the major land covers, uses and associated activities relevant to sub-Saharan Africa, while being broadly comparable with other land-use maps (e.g., Ellis *et al.*^40^; Goldewijk *et al.*^41^; Hurtt *et al.*^42^). Experts made estimates at a ‘landscape’ scale (i.e., several square kilometres). A finer ‘patch’ scale (i.e., several square metres) would be inappropriate for an expert-elicited approach because experts need to consider multiple activities in an area that impact species. For example, a patch of vegetation in a city is likely to have a different impact on a species than a patch of vegetation in a smallholder cropland or a protected area (particularly for large-bodied or wide-ranging species). Instead, the landscape of land covers, uses and activities characteristic of each land use category were described to experts (Table 2), who were asked to consider the collective impact on the abundance of a given group of species within that land use^28^.

Experts were provided with photographic examples of these land uses to help visualise them and promote consistency in scoring between experts (Supplementary Table 2). For each land use, experts were asked to visualise a specific landscape with which they are familiar that matched the description provided, or several such landscapes across which they could consider an average. Experts were instructed to consider the integrated impact of all characteristics of that landscape (e.g., habitat loss and fragmentation, land cover change, disturbance by people, their domestic animals, infrastructure, pollution, harvesting, persecution, introduced species and diseases, elimination of mutualistic species, altered fire and herbivory regimes) on each species response group. Integrated information at a landscape scale is also likely to be more useful for certain decision-maker needs than fine-scale, species-level data^23^.

### Structured expert elicitation

**Fig. 3:**
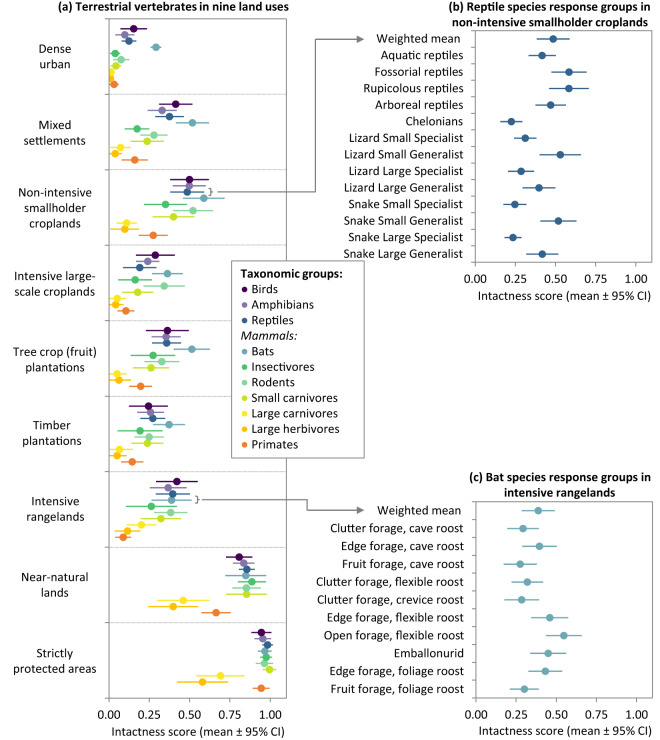
Intactness scores depicting the remaining proportions of ‘intact’ reference populations of terrestrial vertebrates (tetrapods) in different land uses, where 0 indicates that no individuals remain and 1 indicates the same number of individuals as in an ‘intact’ reference population. Average scores across experts (±95% confidence intervals; CI) are shown. The left panel (**a**) depicts an aggregated score for each taxonomic group and land use – an average across species response groups, weighted by species richness (i.e., response groups representing a higher number of species in a taxonomic group count more towards its aggregated score). The right panels show examples of the scores for species response groups in two taxonomic groups in different land uses: (**b**) reptiles in non-intensive, smallholder croplands and (**c**) bats in intensive rangelands.

The IDEA (‘Investigate’, ‘Discuss’, ‘Estimate’, and ‘Aggregate’) structured expert elicitation protocol^25^ was used (Fig. 1). It is a modified-Delphi procedure that treats each step as a process of formal data acquisition, incorporating research from mathematics, psychology, and decision theory to help reduce the influence of biases and enhance the transparency, accuracy, and repeatability of the resulting estimates^25^. The protocol was implemented as follows for each of the broad taxonomic groups:

1. Investigate: A one-hour online meeting was held to introduce the project and explain what was expected of participating experts. Experts had an opportunity to ask questions. The lead expert also presented the draft species response groups and received feedback from the participating experts that, in many cases, led to revisions of these groupings. Afterwards, experts were emailed a recording of the meeting, written instructions, and a survey spreadsheet in which to provide estimates. Experts could provide estimated intactness scores for all species response groups (ranging from six groups for primate experts to 33 for plant experts; Table 1) in all nine land uses (Table 2) or for a subset of species response groups and land uses, depending on the extent of their knowledge. Experts were encouraged to provide any comments relevant to each estimate (e.g., land use characteristics that could influence their score, assumptions that they made, uncertainties, the likely score range across species in a group, and any other explanatory information). Experts could also add general reflections to a comments box at the bottom of the spreadsheet. This qualitative information was useful when aggregating the data (step 2), to gain insight into the reasoning behind experts’ scoring, and detect potential inconsistencies between experts. Experts were asked not to talk to other participants about their estimates to ensure that they did not influence each other’s initial scoring. Experts were encouraged to use other means available to them to inform their scoring, such as talking to colleagues, consulting literature and relevant species reference lists, drawing on experience, and acquiring and interpreting data. They were given two weeks to email their ‘Round 1’ spreadsheet to the project lead.
2. Discuss: A one to 1.5-hour online meeting was convened, where aggregated (anonymised) results from Round 1 were presented to participating experts. These aggregated results included boxplots showing the range of estimates that experts provided for each response group and land use (and biome, for plants), and 95% confidence interval plots showing trends in mean intactness scores across experts for each response group–land use combination (see examples in the right-hand panels of Figs. 3, 4). Project and expert leads reflected on key trends and sources of variability, and any insights or discrepancies (e.g., when it was apparent from experts’ spreadsheet comments that they were interpreting a given land use in different ways). The project lead then facilitated a discussion among the experts, where they were encouraged to share their experiences from Round 1 (e.g., with what did they struggle; what helped them) and to reflect on the aggregated results (e.g., their insights for the species response groups they know well, or any results that surprised them). They were encouraged to discuss outlying (anonymous) expert estimates and why they may have occurred. The project lead emphasised that the purpose of the discussion was not to reach consensus. Rather, it was to interrogate sources of variability, improve the consistency with which experts were interpreting the species response groups and land uses, and cross-examine reasoning, assumptions and evidence, thereby sharing insights between experts to promote learning^25^. (We found this discussion meeting to be a particularly useful step in the process, with many experts providing feedback saying that they found it an enjoyable opportunity to learn from each other.)

**Fig. 4:**
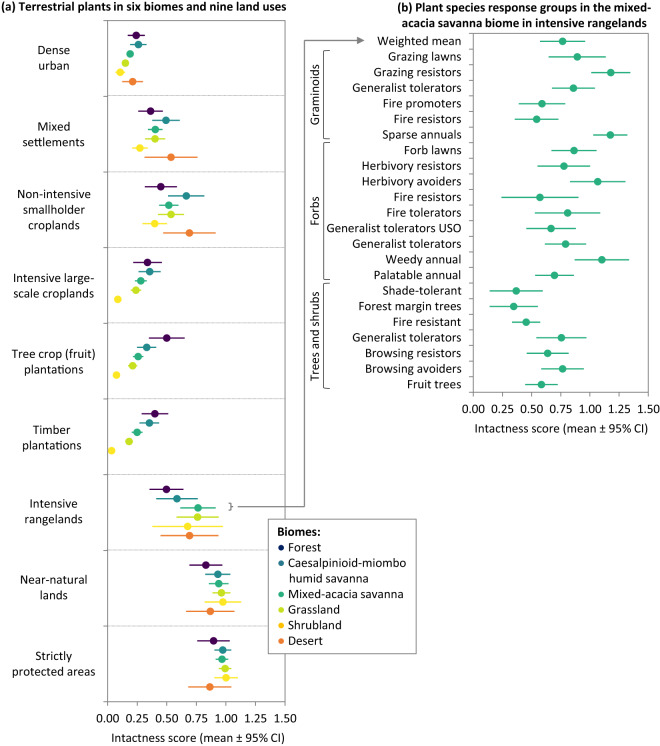
Intactness scores depicting the remaining proportions of ‘intact’ reference populations of terrestrial vascular plants in different land uses, where 0 indicates that no individuals remain and 1 indicates the same number of individuals as in an ‘intact’ reference population. Average scores across experts (±95% confidence intervals; CI) are shown. The left panel (**a**) depicts an aggregated score for plants in each land use in each biome – an average across species response groups, weighted by species richness (i.e., response groups representing a higher number of species in a biome count more towards its aggregated score). The right panel (**b**) shows an example: the scores for plant species response groups in intensive rangelands in the mixed-acacia savanna biome. (Thicket and fynbos biomes are not shown because of low sample sizes: <3 expert scores across all land uses; USO = underground storage organ).
3. Estimate: An email was sent to participating experts with instructions for Round 2 of the elicitation. This email included a recording of the discussion meeting, written summary of key points, and the Round 1 aggregated results plots. Based on the meeting discussion and summary, experts were asked to revisit their initial scores and independently revise any of these scores if they deemed it necessary (again without discussing their individual scores with other participants). It was emphasised that the objective was not to revise their scores to be closer to the ‘mean’, but rather to revise scores if the discussion gave the expert additional insight that caused them to reconsider their initial estimates (which may or may not result in their revised scores being closer to the mean). Experts had one week to either send revised estimates to the project lead, or to confirm that they did not need to make any revisions.
4. Aggregate: Final ‘Round 2’ estimates were aggregated by calculating the mean expert score and confidence interval across expert scores, for each species response group and landscape (and biome for plants). These final confidence interval plots were shared with experts via email. The average change from Round 1 to 2 in variation between expert estimates was assessed (see *Technical Validation*). The resulting dataset of intactness scores^35^ includes both individual experts’ Round 2 estimates and associated comments (‘Scores_Raw’ spreadsheet) and aggregated scores across experts (‘Scores_Agg’ spreadsheet).

## Data Records

The bii4africa dataset is presented in a multi-spreadsheet .xlsx (Microsoft Excel Spreadsheet) file, which is freely accessible in Figshare^35^. The raw data spreadsheet (‘Scores_Raw’) includes 31,313 individual expert estimates of the impact of a sub-Saharan African land use (Table 2) on a species response group of terrestrial vertebrates or vascular plants (Table 1). Estimates are reported as intactness scores – the remaining proportion of an ‘intact’ reference (pre-industrial or contemporary wilderness area) population of a species response group in a land use, on a scale from 0 (no individuals remain) through 0.5 (half the individuals remain), to 1 (same as the reference population) and, in limited cases, 2 (two or more times the reference population). For species that thrive in human-modified landscapes, scores could be greater than 1 but not exceeding 2 to avoid extremely large scores biasing aggregation exercises. Such truncation is common in standardised biodiversity metrics^18,28,43^. Expert comments are included alongside respective estimates.

The raw dataset links, via unique species response group codes, to a spreadsheet (‘Sp_Groups’) describing the species response groups for which experts provided intactness scores (see summary in Table 1). For terrestrial vertebrates, the response group codes also link to a spreadsheet (‘Sp_Vert’) containing all IUCN-listed species in sub-Saharan Africa. Each species has been assigned to one species response group. As large herbivores were initially allocated to response groups but ultimately scored at the species level (see *Identifying and describing species response groups* section), each species has been assigned the appropriate response group code, with a unique letter differentiating species (e.g., African elephant and black rhinoceros, both in the ‘megaherbivore’ response group LH1 in Supplementary Table 1, are assigned codes LH1A and LH1B, respectively). For terrestrial vascular plants, the response group codes link to a spreadsheet (‘Sp_Plant’) containing aggregated expert-elicited estimates of the proportion of species in each biome that constitute each response group, within the broad groups of trees and shrubs, forbs and graminoids. Biome names also link to a spreadsheet (‘Biomes’) that includes a list of the 89 mainland Afrotropical ecoregions^36^, each allocated to one biome (or two biomes if considered a mosaic).

The raw dataset links via unique, anonymous expert codes to a spreadsheet (‘Experts’) listing the region(s) of expertise of each expert (Central Africa, East Africa, Horn of Africa, Southern Africa, West Africa). A summary of the attributes of the 200 participating experts (including their regions of expertise) is provided in Fig. 2. Expert codes are also linked to a spreadsheet (‘Comments’) containing general comments made by experts. The number of intactness scores provided by each expert (‘Scores_Raw’) varied based on the elicitation that they participated in (e.g., primate experts were asked to score six species response groups, while bird experts were asked to score 17 such groups, see Table 1) as well as their extent of expertise (i.e., some experts provided scores for only a subset of species response groups and/or land uses). On average, each expert provided 155 intactness scores.

The number of experts estimating an intactness score for a given species response group in a given land use (and biome for plants) varied from one to 28, with an average (±SD) of ten (±7) experts providing independent scores for a particular combination. Arithmetic means are the most widely used form of data aggregation in applications of the IDEA expert elicitation protocol^25^. For each species response group in each land use (per biome for plants), we report the mean intactness score across experts, the number of experts providing a score (sample size), as well as the variability in scores between experts (standard deviation, standard error, 95% confidence interval; ‘Scores_Agg’ spreadsheet). Figs. 3, 4 visualise the data at two levels of aggregation. The right-hand panels display examples of the aggregated (‘Scores_Agg’) data for reptiles and bats (Fig. 3) and mixed acacia-savanna plants (Fig. 4). These plots depict the variation in how different species response groups within a given taxonomic group are expected to be impacted by a given land use (differences between response group mean scores). They further depict variation between estimates of individual experts (95% confidence intervals around each response group mean score).

The left-hand panels in Figs. 3, 4 display a further level of data aggregation, in which intactness scores for each taxonomic group in each land use are presented. Each score is an average across the associated species response group means and confidence intervals (‘Scores_Agg’), weighted relative to the proportion of species in that response group (‘Sp_Vert’ and ‘Sp_Plant’). In other words, each species in sub-Saharan Africa counts equally in the aggregated intactness score. Such an aggregation could similarly be performed for all species in a given ecoregion, country, or other spatial unit. The dataset can be downloaded together with an R code script for performing such aggregations.

Notably, the ‘strictly protected areas’ land use does not always have an intactness score of 1 (i.e., equivalent to a pre-industrial ‘intact’ reference population; Figs. 3, 4). These lower scores are because (a) some species benefit from human-related disturbances that would have been present in a pre-industrial landscape but are no longer present in a strictly managed protected area and (b) for particularly large, wide-ranging species, even the best-managed protected area is unlikely to contain a ‘pre-industrial’ reference population (e.g., Prins *et al*.^44^; Balme *et al*.^45^). It is also worth noting that not all protected areas in the region are strictly protected in practice – these protected areas were not considered in this land use category, and intactness scores for such protected areas would likely be closer to the ‘near-natural lands’ land use or even some of the other land use categories (e.g., intensive rangelands or non-intensive smallholder croplands; Table 2), depending on the activities occurring in these areas.

## Technical Validation

The reliability of expert judgement will always be sensitive to which experts participate and how questions are asked. Each step of the IDEA structured expert elicitation protocol makes use of procedures that have been demonstrated to improve elicitation rigour^25^. These include the identification and recruitment of experts, the framing of questions, the two rounds of independent estimation and the aggregation, review and critical appraisal of expert judgements during a facilitated group discussion (see *Methods*).

**Fig. 5:**
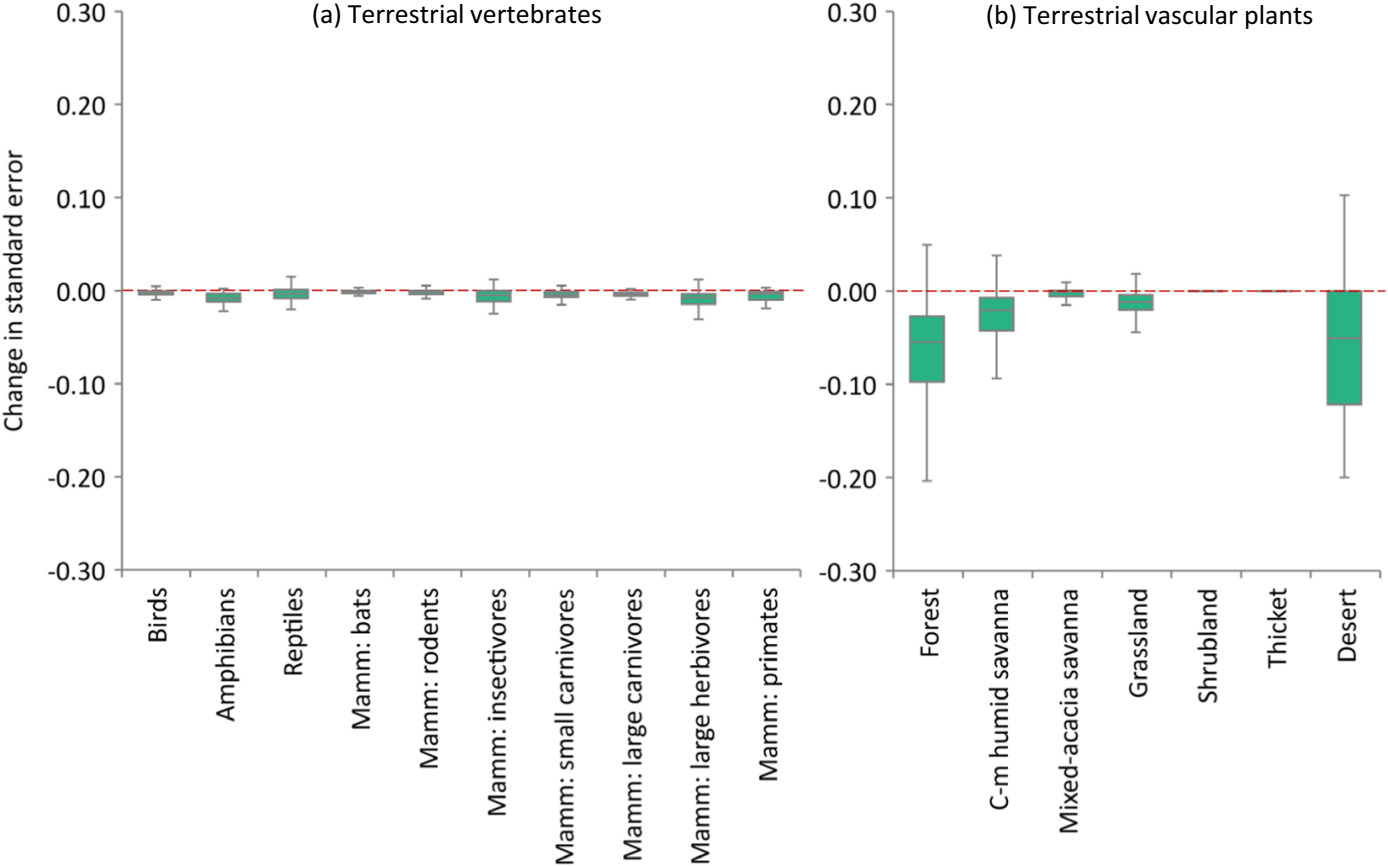
Change in the variation (standard error) of estimated intactness scores for each species response group and land use, between the first and second round of the expert elicitation process. Boxplots show median (horizontal line in the box), interquartile range (box), and max/min values within 1.5 times the interquartile range (vertical lines). Values less than 0 (below the red-dashed horizontal line) show a decrease in score variability between experts. For terrestrial vertebrates, results are shown per taxonomic group; for terrestrial vascular plants they are shown per biome. (C-m = Caesalpinioid-miombo; Mamm = Mammals).

The purpose of the facilitated group discussion is not to reach consensus^25^. In our case, experts could disagree on how species are likely to respond to land uses, particularly for lesser-known species groups. Rather, the discussion in a modified-Delphi process aims to resolve linguistic ambiguity, promote critical thinking, share evidence, and improve the consistency with which experts interpret the questions^25^. Thus, we would expect some but not all of the variability in expert scores to reduce in the second round of the elicitation process, subsequent to the group discussion. The standard error around expert scores for each species response group in each land use (and in each biome for terrestrial vascular plants) was lower in the second, compared with the first, round of the elicitation for 85% and 88% of terrestrial vertebrate and plant intactness scores respectively (Fig. 5). On average, there was a reduction in standard error of 0.01 for vertebrates and 0.03 for vascular plants (Fig. 5). Thus, the variability in estimates between experts was generally lower following the group discussions, indicating that the elicitation process resulted in improved scoring consistency between experts.

We would also expect a reasonable degree of consensus between experts, and therefore for the variability between their scores to be significantly lower than that of chance. To test this expectation, we generated a random estimate within the allowed range (0 to 2) for each Round 2 expert estimate. We then determined the standard error around these random estimates for each species response group and land use (and biome for terrestrial vascular plants). This process ensured comparable sample sizes between expert estimates and random estimates. The standard error in expert estimates was significantly lower than that expected by chance (paired t test: *t* = −54.58, *d.f*. = 2796, *p* < 0.001). On average, the standard error in expert estimates in Round 2 was 63% lower than expected by chance (0.075 compared with 0.200). These results suggest that (1) the IDEA protocol served to promote consistency in scoring between experts and (2) experts were significantly more consistent in their scoring than expected by chance.

Validating this dataset using available field-collected data is limited by a spatial and temporal scale mismatch. Most field data are collected at the ‘patch’ scale, while the landscape scale was appropriate for producing this dataset, as explained in the methods. The experts considered diverse land covers and activities characteristic of each land use type (Table 2) to estimate their collective impact on a population in that land use. For example, in an agricultural landscape there is likely to be a higher abundance of fossorial reptiles in the habitat remnants than in the surrounding croplands. This landscape composition was considered by experts when estimating the overall remaining proportion of a reference population of these reptiles in a landscape characterised by non-intensive, smallholder croplands (or intensive, large-scale croplands, with fewer remnant habitat patches). In contrast, field studies tend to report land use at the scale of the habitat or cropland patch. For example, in the PREDICTS database^22^—the largest global dataset of species abundances in different land uses—the only relevant cropland data^46^ for Afrotropical birds contains bird counts in ‘private farmhouse gardens surrounded by agricultural matrix’. This ‘patch’ scale, focused on a small subset of land covers and activities in an agricultural landscape, is incompatible with the cropland landscapes that experts considered (Table 2). The spatial and temporal scale of the reference site can also be mismatched. For example, the only relevant plantation data^47^ for Afrotropical birds in the PREDICTS database has a reference site of ‘forest fragments in timber plantations’, which is incompatible with the landscape-scale pre-industrial/large wilderness area reference state that experts considered in this study. While this scale mismatch limits validation using existing field data across multiple regions and taxa, future research aimed at validation could design landscape-scale data collection protocols that are more comparable (i.e., developing landscape-scale data collection protocols with multiple multi-taxa sampling sites evenly distributed across different land uses across the region). However, the absence of a ‘pre-industrial’ reference in field-collected data can still impede comparison^17^.

## Usage Notes

A non-exhaustive list of potential uses of the bii4africa dataset is provided in Table 3. The data are best suited to broad-scale, multi-species applications, rather than finer-scale applications for which site-specific, field-collected data would be more appropriate. The standardised nature of the intactness scores (0–2 scale) means that the data (or a subset thereof) can be aggregated in several ways to meet a user’s needs, e.g., by taxonomic group, functional type, land use and/or spatial (e.g., biogeographical or political) unit (see R code provided for aggregating the data^35^). To assist with data aggregation, scores for response groups can be linked back to individual species for terrestrial vertebrates (noting that large mammal scores are already at species level). Spatial distributions of these species are available from the IUCN Red List^26^. A species list can thus be obtained for the area of interest, and those species scores then extracted from the bii4africa dataset. While the scores for terrestrial vascular plants are not connected to individual species, scores are provided per biome, meaning the data can be extracted at biome-scale, informed for example by the WWF ecoregion maps^36^. The proportions of terrestrial vascular plant species in each response group per biome are also provided, enabling the weighting of score aggregations relative to the proportion of species that those scores represent. Data can also be aggregated according to the regional expertise of the contributing experts (e.g., using scores from only West African experts for a West African data application, or for testing differences between regions). For some species groups such as large mammals and birds, regional considerations such as whether bushmeat harvesting is prevalent can have an influence on experts’ intactness scores.

While the data presented here are non-spatial, they can be made spatially explicit by linking the scores for different land uses to a map of those land uses (encompassing sub-Saharan Africa or a region therein). As the land uses were selected to reflect those most common in sub-Saharan Africa, the map used to spatialise the data should include those classes (urban, crop, plantation, rangeland, near-natural, protected). See Scholes and Biggs^28^, Newbold *et al*.^29^ and Schipper *et al*.^18^ for examples of mapping Biodiversity Intactness based on intactness scores for different land uses. Different land use intensities (e.g., dense urban vs mixed settlements; smallholder vs large-scale cropland; rangeland vs near-natural land) could also be mapped using proxies such as percentage urban cover^48^ and population density^49^ in human settlements; percentage crop cover^50^, nitrogen input^51^ and field size^52^ in croplands; livestock density^53^ in rangelands; etc. Importantly, as land use changes across the region, estimates and maps of intactness can be updated using the bii4africa dataset.

Following the IDEA protocol recommendations^25^, outliers were not removed from the data when determining mean intactness scores across experts (‘Scores_Agg’ spreadsheet in the dataset). Rather, anonymised outliers were flagged in the discussion meeting, after which experts who provided such scores could reconsider if they were appropriate and revise them if not. Equally weighted data aggregations (i.e., arithmetic means) can be sensitive to outliers in small groups, and we thus recommend careful consideration regarding the use of mean scores that are based on a low number of experts. This consideration is most relevant for large mammals and terrestrial vascular plants – the only groups with mean scores for some species (large mammals) or species response groups (plants) in some land uses based on fewer than the recommended six experts^25^. With an average of 10 contributing experts per mean score, our dataset has a considerably larger ‘sample size’ than other similar processes (e.g., n = 3)^28^. We report sample size and standard deviation in our aggregated dataset, as well as provide the raw data, enabling users to assess whether the degree of variability in scores is acceptable for their purposes. We also think the scores with higher variability (i.e., less consensus between experts) could identify important knowledge gaps regarding how species respond to land uses, or important regional differences, thus informing future empirical research.

### Supplementary information

Supplementary Table 3

Supplementary Table 2

Supplementary Table 1

## Data Availability

R code for calculating aggregated intactness scores for a focal region (e.g., ecoregion or country) and/or taxonomic group can be downloaded with the bii4africa dataset on Figshare^35^; see Data Records section.
