# Supplementary material for: The bii4africa dataset of faunal and floral population intactness estimates across Africa’s major land uses
Source: Sci Data. 2024 Feb 12;11:191. doi: 10.1038/s41597-023-02832-6 (PMC10861571; doi:10.1038/s41597-023-02832-6)
Supplement: Supplementary file 1 — Supplementary Table 3 [file 41597_2023_2832_MOESM1_ESM.pdf]

Supplementary Table 3. A non-exhaustive list of potential uses of the bii4africa dataset.

Use	Details
Quantifying ecosystem integrity / condition across space and through time	The first goal of the post-2020 Global Biodiversity Framework is to increase the 'area, connectivity and integrity of natural ecosystems', where integrity is defined as 'the compositional functional, structural and spatial components of ecosystems'. Limited data are available for assessing ecosystem integrity (often also referred to as ecosystem condition), with simple metrics such as habitat loss often used as proxies. South Africa, a regional leader in biodiversity mapping, states in its National Biodiversity Assessment Report that 'Habitat loss is a simple measure of ecological condition that is reliably collected using land cover change datasets, however, there is a major gap in our ability to measure the subtler forms of habitat modification and estimate ecosystem condition'[1]. This gap is particularly problematic for land uses that do not correspond clearly with a transformed land cover, such as rangelands – one of the largest land uses in Africa. The dataset can be used to translate land use extents into subtler forms of ecosystem condition, such as through mapping the Biodiversity Intactness Index[2] and monitoring its change over time.
Assessing the severity of functional decline for the IUCN Red List of Ecosystems	The IUCN Red List of Ecosystems is a tool for classifying threatened ecosystems, informing ecosystem management and assessing the risk of ecosystem collapse. However, these risk assessments require explicit definitions of ecosystem collapse, and measures of the severity of functional decline, which are challenging to implement[3]. Aggregated indices of ecosystem health or condition are proposed as one option for quantifying functional decline[4]. The dataset could enable such a quantification. Trends in functional decline that emerge from such a quantification could similarly be used to guide the development of thresholds of potential concern / thresholds for management intervention[5].
Quantifying relative population abundance and biodiversity composition indicators	Several composite biodiversity indicators include a measure of population abundance relative to a reference state, often within a biodiversity composition category, including Essential Biodiversity Variables[6]; Multidimensional Biodiversity Index[7]; Ecosystem Integrity Index[8,9]; Biodiversity Intactness Index[2,10]; and Mean Species Abundance metric (GLOBIO)[11,12]. Several of these are also proposed indicators in the Global Biodiversity Framework. The dataset could be used to inform such indicators, alleviating taxonomic and regional biases in existing indicators. If coupled with data / predictions of absolute population abundance in a reference population (e.g., Hudson et al.[13]; Santini et al.[14,15]), relative abundances (i.e., intactness scores) could also be translated into indicators of absolute abundance.
Setting conservation and restoration goals and/or monitoring progress towards these goals	We are now in the UN Decade on Ecosystem Restoration, which aims to halt the degradation of ecosystems, and restore them to achieve global goals. Restoration and conservation initiatives often require a 'target' to work towards – a challenge in the absence of an appropriate reference state / baseline[16]. The dataset could be used to assess progress towards restoring 'intactness' in a region (e.g., if the intactness score for a given species group in an intensive rangeland is 0.5, then the ultimate target for restoration efforts is to double that population, something that could be monitored over time). The data could also be used in prioritisation exercises to identify ecosystems for restoration action to maximise improvements in biodiversity intactness.
Assessing the impact of regional development plans	Large-scale infrastructure and agriculture projects are planned across sub-Saharan Africa (e.g., Laurance et al.[17]). This dataset could be used to predict the impacts of such development plans on biodiversity intactness.
Considering biodiversity sensitivity to development	The data could identify the types of taxa that are particularly sensitive to development, to inform Environmental Impact Assessments and other development plans. If the dataset is spatialised, it could also be used to identify ecosystems that are particularly sensitive to development.
Identifying indicator species groups	The data could be used to identify indicator species groups within particular regions or land uses. Species groups with lower intactness scores are more vulnerable to environmental or developmental change, and monitoring their populations could give early warnings of system degradation or be used as sentinel species groups in impact assessments. Similarly, conservation efforts aimed at protecting these vulnerable species groups should simultaneously conserve a variety of other species.

Assessing trends in how diverse species respond to land use activities	The data could be analysed to test hypotheses and explore trends across species groups and/or land uses. Many aggregated biodiversity indices are biased towards large mammals and birds, and the dataset could be used to assess the implications of such biases, by comparing intactness trends for these groups to those for lesser studied groups (e.g., amphibians). It could also be used to explore synergies and trade-offs between land uses for different types of species with diverse attributes and functions. Such assessments could guide decisions around land management and conservation efforts.
Species ecological (as opposed to taxonomic) classifications	The species response groups presented in this dataset (Table 1) can be considered hypotheses from a collection of experts on the major attributes that influence species' population responses to human-modified landscapes. These groupings may be useful for a range of applications that require species to be organised into 'functional' (as opposed to purely taxonomic) categories, or to allow data-rich species to serve as proxies for data-poor species in a category. Future empirical research could also test these hypotheses.
Zoonotic disease risk and mitigation assessments	The dataset could be used in identifying and monitoring species groups (and areas, if spatialised) to prevent zoonotic and epizootic disease outbreaks if combined with knowledge on zoonoses in species that commonly overlap with densely populated human areas (or other land uses where the interaction between humans and wildlife may be expected to increase or change). It could be used to forecast spill-over risks of zoonotic diseases in different land-use types by understanding how reservoir host groups (bats, rodents, small carnivores, etc.) respond to anthropogenic environmental change.
Characterising novel ecosystems	Intactness scores >1 depict species groups that respond positively to human land use activities, thus contributing to novel ecosystems. The dataset could be used to quantify the degree and type of 'novelty' in a region – a categorisation that may be useful for informing studies of novel ecosystems[18].
Parameterising, calibrating and validating models of biodiversity in a changing world	Biodiversity models (mechanistic or correlative) are used to predict biodiversity patterns across space and through time (e.g., Di Marco et al.[19]; Harfoot et al.[20]; Schipper et al.[11]) under changing land use conditions (which may also be affected by climate change). This dataset could be used to parameterise, calibrate, or validate such models. It could also be used to identify species groups or ecosystems that are threatened by projected changes versus those that will cope well. Thus, it lends itself to the development of predictions/hypotheses of how species may respond to future changes in land-use extent and intensity, and to identify major threats.
Climate change research	Given the focus of this dataset on land use impacts, it examines biodiversity across a microhabitat mosaic. The effects of climate change may be expected to vary across this mosaic. Therefore, the approach taken in this paper offers opportunities for natural and/or experimental designs to test interactions of biodiversity, land use and climate change across variable spatial and temporal scales.
Informing future research and training in biodiversity	The species groups and land uses for which there were either few expert scores or large expert score variability highlight knowledge gaps that require further study. This dataset has the potential to direct future research on the impacts of various land uses on species. These knowledge gaps could also be used to guide future scientist training efforts in taxonomy, ecology, biogeography, and conservation.
Comparison with other regions, taxonomic groups or time periods	A similar expert-elicited approach could be used to estimate intactness scores for other regions in the world and/or other taxonomic groups (e.g., invertebrates), allowing for comparison with this dataset. The same approach could also be undertaken again in the future to assess how knowledge on land use impacts on biodiversity abundance has changed.
List of biodiversity experts to contact for data, collaboration, etc	The 200 participating experts (see author list and contributions, and https://bii4africa.org/category/experts/) can serve as points of contact for global initiatives looking to aggregate data or build collaborations.

References

1. Skowno, A. L., Raimondo, D. C., Poole, C. J., Fizziotti, B. & Slingsby, J. A. *South African National Biodiversity Assessment 2018 Volume 1: Terrestrial Realm*. *South African National Biodiversity Institute* (2019).
2. Scholes, R. J. & Biggs, R. A biodiversity intactness index. *Nature* **434**, 45–49 (2005).
3. Bland, L. M. *et al.* Developing a standardized definition of ecosystem collapse for risk assessment. *Front. Ecol. Environ.* **16**, 29–36 (2018).
4. Keith, D. A. *et al.* The IUCN red list of ecosystems: Motivations, challenges, and applications. *Conserv. Lett.* **8**, 214–226 (2015).
5. Hilton, M., Walsh, J. C., Liddell, E. & Cook, C. N. Lessons from other disciplines for setting management thresholds for biodiversity conservation. *Conserv. Biol.* **36**, 1–15 (2022).
6. Jetz, W. *et al.* Essential biodiversity variables for mapping and monitoring species populations. *Nat. Ecol. Evol.* **3**, 539–551 (2019).
7. Soto-Navarro, C. A. *et al.* Towards a multidimensional biodiversity index for national application. *Nat. Sustain.* **4**, 933–942 (2021).
8. Hill, S. L. L. *et al.* The Ecosystem Integrity Index: a novel measure of terrestrial ecosystem integrity with global coverage. *bioRxiv* 2022.08 (2022).
9. Hansen, A. J. *et al.* Towards monitoring forest ecosystem integrity within the Post-2020 Global Biodiversity Framework. *Conserv. Lett.* **14**, e12822 (2021).
10. Newbold, T. *et al.* Has land use pushed terrestrial biodiversity beyond the planetary boundary? A global assessment. *Science* **351**, 600–604 (2016).
11. Schipper, A. *et al.* Projecting terrestrial biodiversity intactness with GLOBIO 4. *Glob. Chang. Biol.* **26**, 760–771 (2020).
12. Alkemade, R. *et al.* GLOBIO3: A framework to investigate options for reducing global terrestrial biodiversity loss. *Ecosystems* **12**, 374–390 (2009).
13. Hudson, L. N. *et al.* The database of the PREDICTS (Projecting Responses of Ecological Diversity In Changing Terrestrial Systems) project. *Ecol. Evol.* **4**, 4701–4735 (2016).
14. Santini, L., Isaac, N. J. B. & Ficetola, G. F. TetraDENSITY: A database of population density estimates in terrestrial vertebrates. *Glob. Ecol. Biogeogr.* **27**, 787–791 (2018).
15. Santini, L., Benítez-López, A., Dormann, C. F. & Huijbregts, M. A. J. Population density estimates for terrestrial mammal species. *Glob. Ecol. Biogeogr.* **31**, 978–994 (2022).
16. McNellie, M. J. *et al.* Reference state and benchmark concepts for better biodiversity conservation in contemporary ecosystems. *Glob. Chang. Biol.* **26**, 6702–6714 (2020).
17. Laurance, W. F., Sloan, S., Weng, L. & Sayer, J. A. Estimating the Environmental Costs of Africa's Massive 'Development Corridors'. *Curr. Biol.* **25**, 3202–3208 (2015).
18. Hobbs, R. J., Higgs, E. & Harris, J. A. Novel ecosystems: implications for conservation and restoration. *Trends Ecol. Evol.* **24**, 599–605 (2009).
19. Di Marco, M. *et al.* Projecting impacts of global climate and land-use scenarios on plant biodiversity using compositional-turnover modelling. *Glob. Chang. Biol.* **25**, 2763–2778 (2019).
20. Harfoot, M. B. J. *et al.* Emergent Global Patterns of Ecosystem Structure and Function from a Mechanistic General Ecosystem Model. *PLoS Biol.* **12**, e1001841 (2014).
