# Supplementary material for: The bii4africa dataset of faunal and floral population intactness estimates across Africa’s major land uses
Source: Sci Data. 2024 Feb 12;11:191. doi: 10.1038/s41597-023-02832-6 (PMC10861571; doi:10.1038/s41597-023-02832-6)
Supplement: Supplementary file 2 — Supplementary Table 2 [file 41597_2023_2832_MOESM2_ESM.pdf]

Supplementary Table 2. Descriptions along with representative images of the nine sub-Saharan African land uses presented to experts to estimate the remaining proportion of an 'intact' reference population, for diverse groups of species. (Image source: Unsplash.com)

Land use	Description
Dense urban	Densely built-up environments with high human population densities and limited green space — city centres, dense townships, industrial areas, transformed mining areas (e.g., open cast mines, quarries, dumps). Most ecological processes are highly modified. There are few remaining near-natural patches in the landscape, except for e.g., road-side trees and small parks.<br>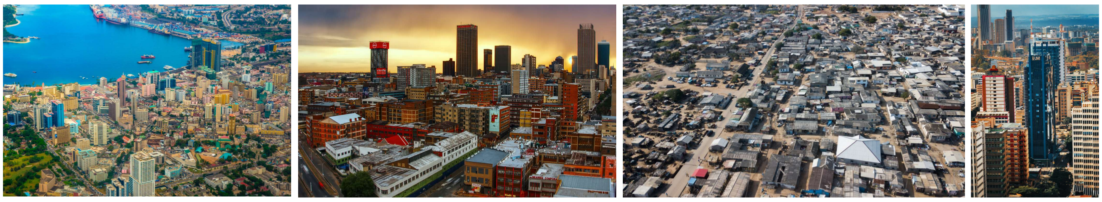
Mixed settlements	Suburban areas, smaller towns and rural settlements with large but fragmented human populations interspersed with gardens, parks and near-natural patches of open space, potentially with low densities of cattle, goats, sheep or chickens, or small-scale croplands.<br>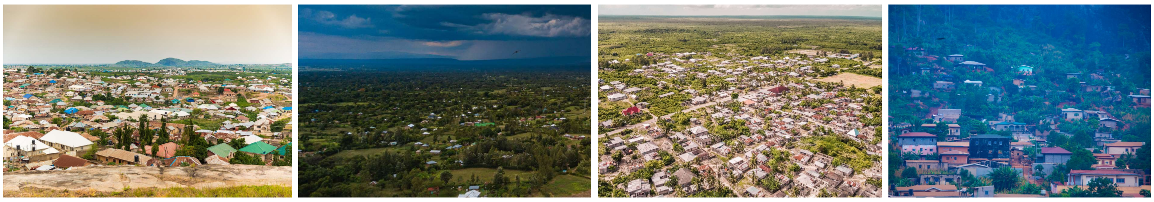
Non-intensive smallholder croplands	Lands used mainly for smallholder agriculture in small fields (<2 ha), consisting of a diversity of short-duration and long-duration crops (e.g., maize, millet, cassava, beans, squashes, as well as scattered fruit, shade or timber trees). Agricultural inputs of fertilisers and pesticides are very low if any, cultivation is usually manual, there is little or no ploughing or irrigation, and harvest is staggered in time. Fields and homesteads are interspersed with patches of near-natural vegetation. These lands often also support low densities of livestock or smallstock, which are partly free-roaming, and may have semi-natural grazing areas in addition to eating crop residues and cut forage.<br>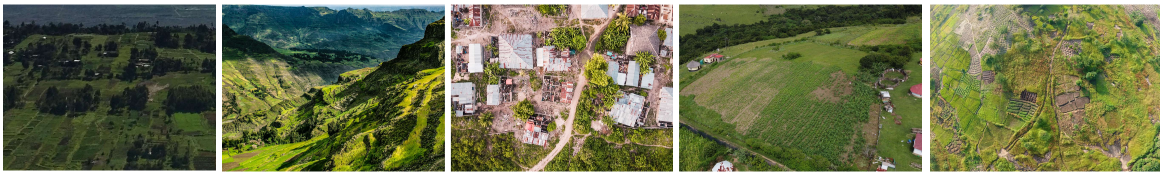
Intensive large-scale croplands	Lands used mainly for short-duration, monocultural crops in large fields (e.g., staple cereal crops, soybeans, sugar cane). Land use activities usually include several of the following: annual ploughing, inorganic fertiliser application, pesticide application, irrigation, mechanisation. When the crop is harvested, the entire biomass is removed and the next crop is planted, perhaps after a fallow period. There are few remaining near-natural patches in the landscape, except for instance on drainage lines, field boundaries and contour strips, or some woodlots or windbreaks of trees.<br>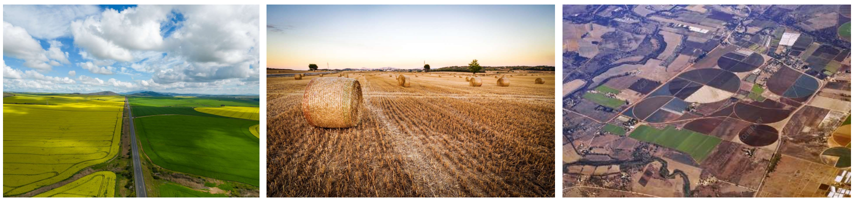

Tree crop (fruit) plantations	Lands used mainly for tree crops including fruit-bearing tree or shrub plantations (e.g., bananas, coffee, oil palm, cacao, oranges, vineyards, nuts). Non-transformational harvest, usually only the fruit is taken, and trees may be replaced at some stage. Includes limited remnant forest, riparian or grassland patches between plantation compartments.
	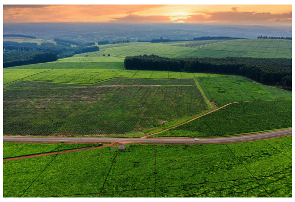 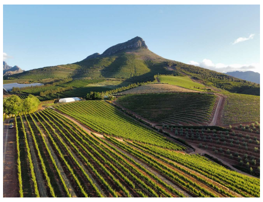 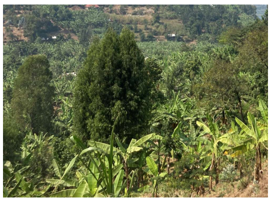 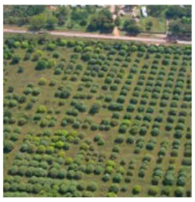
Timber plantations	Lands used for growing trees, typically exotic species, for saw timber, poles or pulp. Harvested by clear-cut every 10 to 30 years, and replanted or regrown from coppice. Includes limited remnant forest, riparian or grassland patches between plantation compartments.
	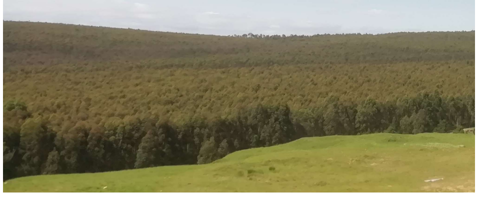 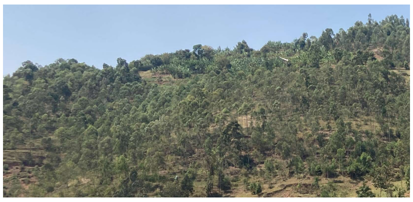 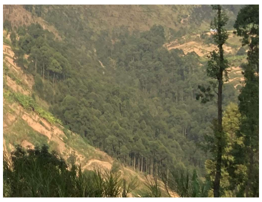
Intensive rangelands	Lands used mainly for livestock grazing either with input of fertiliser or pesticide, or with high stock density relative to what the land can sustain (high enough to cause some disturbance or to stop regeneration of vegetation, or to have done so in the recent past). Domesticated stock such as cattle, sheep, goats are typical, but could also include intensive use of indigenous species such as ostrich.
	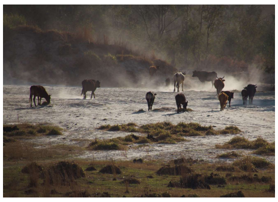 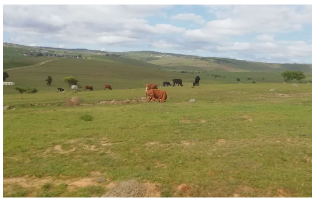 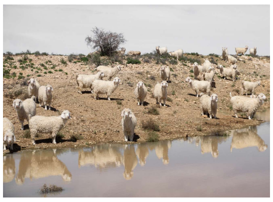 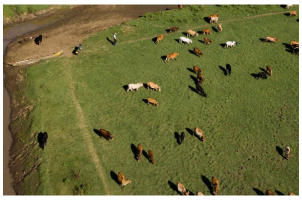
Near-natural lands	Lands (which could be forests, savannas, arid lands, mountainous lands, grasslands) remote from infrastructure, having only minor transformational land use such as crops, planted trees, livestock and human settlements. The human population is relatively low, and livestock or crop-based agriculture or harvest of resources is not at levels that substantially alter natural ecological processes or habitats.
	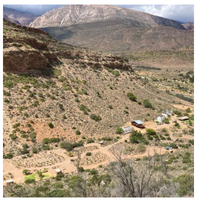 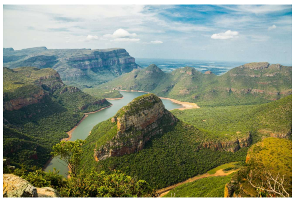 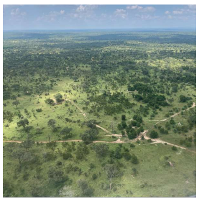 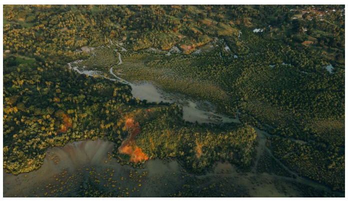
Strictly protected areas	Strictly protected areas that generally do not allow for permanent settlements or resource use, though sometimes allow tourism including limited accommodation and road infrastructure (World Database on Protected Area categories I-III or equivalent). Minimal recent human impact on structure, composition and function of the ecosystem.
	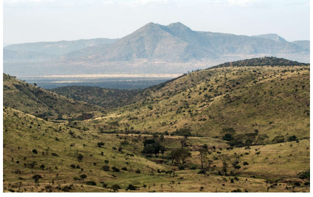 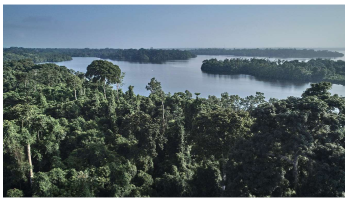 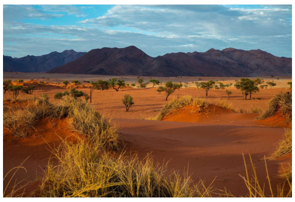
